# Stromal Cell Contribution to Human Follicular Lymphoma Pathogenesis

**DOI:** 10.3389/fimmu.2012.00280

**Published:** 2012-09-05

**Authors:** Frédéric Mourcin, Céline Pangault, Rada Amin-Ali, Patricia Amé-Thomas, Karin Tarte

**Affiliations:** ^1^INSERM, UMR U917Rennes, France; ^2^Université Rennes 1Rennes, France; ^3^Etablissement Français du SangBretagne, Rennes, France; ^4^Service ITeCH, Pôle de Biologie, CHU de RennesRennes, France

**Keywords:** B cells, bone marrow, lymph nodes, cell interactions, stromal cells

## Abstract

Follicular lymphoma (FL) is the prototypical model of indolent B cell lymphoma displaying a strong dependence on a specialized cell microenvironment mimicking normal germinal center. Within malignant cell niches in invaded lymph nodes and bone marrow, external stimuli provided by infiltrating stromal cells make a pivotal contribution to disease development, progression, and drug resistance. The crosstalk between FL B cells and stromal cells is bidirectional, causing activation of both partners. In agreement, FL stromal cells exhibit specific phenotypic, transcriptomic, and functional properties. This review highlights the critical pathways involved in the direct tumor-promoting activity of stromal cells but also their role in the organization of FL cell niche through the recruitment of accessory immune cells and their polarization to a B cell supportive phenotype. Finally, deciphering the interplay between stromal cells and FL cells provides potential new therapeutic targets with the aim to mobilize malignant cells outside their protective microenvironment and increase their sensitivity to conventional treatment.

## B Cell Lymphoma Microenvironment

**Figure 1:**
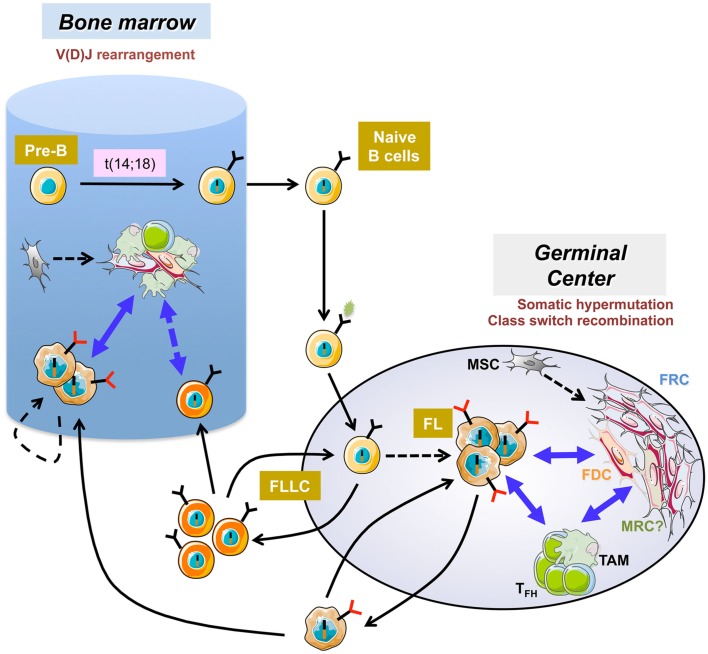
**Follicular lymphoma pathogenesis**. The first step of follicular lymphoma (FL) development occurs in the bone marrow as a mistake in V(D)J rearrangement and leads to the ectopic expression of the antiapoptotic protein bcl2. After antigen encounter, naive B cells harboring the t(14;18) reach the germinal center where they display a selective growth advantage and could extensively recirculate as atypical IgM^pos^ low affinity memory B cells called follicular-lymphoma like cells (FLLC). Iterative reentries into germinal centers allow the acquisition of additional genetic alterations. The relationship between FLLC and FL is not formerly demonstrated but the hypothesis is that FLLC contain premalignant intermediates that could transform in some patients into FL. FL cells remain strongly dependent on bidirectional crosstalk with heterogeneous stromal cells, including activated fibroblastic reticular cells (FRC), altered follicular dendritic cells (FDC), and perhaps marginal reticular cells (MRC) of unknown origin and infiltrating immune cells including tumor-associated macrophages (TAM) and follicular helper T cells (T_FH_). FL also developed in the bone marrow where B-cell clones could evolve independently within ectopic specific FL-supportive niches.

Human mature B cell lymphomas represent a heterogeneous group of neoplasias characterized by recurrent genetic abnormalities and pathway dependencies. Each lymphoma subtype could be assigned to a peculiar stage of normal B cell differentiation, as judged by gene expression profiling and phenotype (Shaffer et al., 2012). In some of them, malignant B cell proliferation, survival, and drug resistance are strongly dependent on a combination of external stimuli delivered by the microenvironment within specific niches in invaded lymph nodes (LN) and bone marrow (BM; Burger et al., 2009). This is particularly true in follicular lymphoma (FL), the most frequent indolent lymphoma, which results from the malignant transformation of germinal center (GC)-derived B cells (Figure 1). Whereas over 90% of FL cases display a *BCL2*/*IGH* translocation, this early event, occurring as a mistake of V(D)J recombination in the BM, could be detected at low frequency within recirculating post-GC memory B cells of most healthy individuals (Roulland et al., 2011). These t(14;18)^pos^ cells exhibit additional characteristics that stand as hallmarks of FL cells; i.e., CD10 expression, unleashed AID activity, persistence of surface IgM despite active class-switch recombination on the translocated allele; are thus called follicular-lymphoma like cells. Given that the actual prevalence of FL is around 0.03%, it is clear that FL pathogenesis requires additional oncogenic events as well as a progressive modification of the composition and organization of tumor microenvironment (Bende et al., 2007). Among the recurrent complementary hits identified in FL patients, several alterations target the transcriptional and epigenetic pathways including inactivation of CREBBP/EP300 acetyltransferases, MLL2 methyltransferase, and MEF2B, involved in the recruitment of histone-modifying enzymes, and gain-of-function mutations of *EZH2* polycomb gene (Shaffer et al., 2012). Recently, a frequent inactivation of the soluble inhibitory receptor EPHA7 was also reported (Oricchio et al., 2011). This inactivation hinders the blockade by EPHA7 of the ephrin migratory pathway induced by cell–cell contact.

In agreement, genetic alterations and modification of the microenvironment are not independent transformation mechanisms since several FL-specific genetic alterations are not oncogenic *per se* but favor specific interactions with neighboring cells. Among them, the frequent mutations of *TNFRSF14/HVEM* could play a role in the maintenance of the functional tumor cell niche (Launay et al., 2012; Pasero et al., 2012). In fact, binding of HVEM to its receptor BTLA delivers an inhibitory signal and BTLA is strongly expressed on FL-supportive follicular helper T cells (T_FH_) within malignant follicles (see below). In addition, nearly all FL-derived immunoglobulin variable regions display unusual sites for N-linked glycosylation, introduced during somatic hypermutation process, and reflecting positive selection associated to lymphomagenesis (Stevenson and Stevenson, 2012). Glycans added to these motifs are atypical in terminating at high mannose that interact with C-type lectins on the surface of surrounding cells and trigger BCR engagement. This functional bridge could mimic for continuous antigen stimulation to promote survival of FL cells (Coelho et al., 2010). About 30% of FL finally transform into aggressive diffuse large B cell lymphomas (DLBCL) that are less dependent on their microenvironment.

Microarray analyses have revealed that the clinical outcome of FL patients is primarily predicted by molecular features of non-malignant cells (Dave et al., 2004). Moreover, immunohistochemical studies have identified a large panel of predictive markers reflecting the number, activation, and/or spatial distribution of infiltrating immune non-B cell subsets, including tumor-associated macrophages (TAM) and CD4^pos^ T cells (Relander et al., 2010). Besides immune cells, three main stromal cell subsets have been described within normal LN: (i) fibroblastic reticular cells (FRC) recruit mature dendritic cells and naïve B and T lymphocytes through the production of CCL19, CCL21, and CXCL12, promote cell–cell interactions within the T cell zone, and are involved in T cell self-tolerance and tissue tropism imprinting; (ii) follicular dendritic cells (FDC) drive CXCL13-dependent B cell attraction within GC where they concentrate unprocessed antigens and promote the selection of high affinity B cells; (iii) marginal reticular cells (MRC) deliver small antigens to cognate B cells through a network of follicular conduits (Mueller and Germain, 2009; Roozendaal and Mebius, 2011). It has long been assumed that some tumor conducive stromal cell niches are shaped within FL LN. In fact, FL LN exhibit a uniform and marked activation of transglutaminase expressing FRC network (Thomazy et al., 2003), whereas FDC display an undifferentiated phenotype (Chang et al., 2003; Jin et al., 2011). MRC have never been evaluated in human lymphomas. More generally a functional characterization of the heterogeneous stromal cell network that is found within malignant follicles is currently lacking. In addition, the *in situ* distribution of lymphoid stromal cell subsets relative to malignant B cells and to the other FL-supporting cell subsets and their activation status in malignant follicles remains unknown.

Follicular lymphoma is generally a disseminated disease and BM is involved in up to 70% of patients at diagnosis (Canioni et al., 2004). BM infiltration is characterized by the ectopic development of heterogeneous lymphoid-like stromal cells of unknown origin that are found admixed with malignant B cells and CD4^pos^ T cells among nodular aggregates (Vega et al., 2002). Even if BM malignant B cells retain their main follicular features, several morphologic, phenotypic, and genetic differences have been reported among tumor cells found within LN and BM. In particular, BM FL cells are characterized by a lower cytological grade and proliferation (Bognar et al., 2005; Rajnai et al., 2012), and exhibit a distinct transcriptomic profile revealing a downregulation of genes involved in cell proliferation and DNA repair (our unpublished data). In addition, despite a common clonal origin, analysis of intraclonal evolution reveals that a significant part of the BM infiltration evolves independently from the tumor clones detected in the LN indicating that BM provides a specific non-lymphoid malignant cell niche (Bognar et al., 2005; Ruminy et al., 2008). Recently, major differences in the cell composition of BM and LN microenvironments have been highlighted (Rajnai et al., 2012; Wahlin et al., 2012) thus paving the way for dedicated studies evaluating the impact of these variations on the behavior of malignant FL cell clones.

Several experimental limitations have hampered a full understanding of the role of stromal cells in FL pathogenesis: (i) the lack of B cell line reflecting the untransformed indolent stage of FL leading to the use of aggressive GC-lymphoma cell lines; (ii) the limited accessibility of tumor biopsies and the high propensity of primary FL cells to undergo apoptosis *in vitro*; (iii) the impossibility to maintain fully functional human FDC in culture; (iv) the heterogeneity of stromal cell subsets that remain poorly understood in human; (v) the inherent flaws of human FL B cell xenotransplantation into immunocompromised mice that are devoid of structured secondary lymphoid organs; (vi) the lack of validated relevant mouse model of FL. Nevertheless, even if these pitfalls should be carefully kept in mind, some recent studies have provided interesting results about lymphoma-permissive stromal cells, revealing both a direct B cell supportive effect and an indirect activity on the orchestration of the FL cell niche.

## Direct Protumoral Activities of Stromal Cells

**Figure 2:**
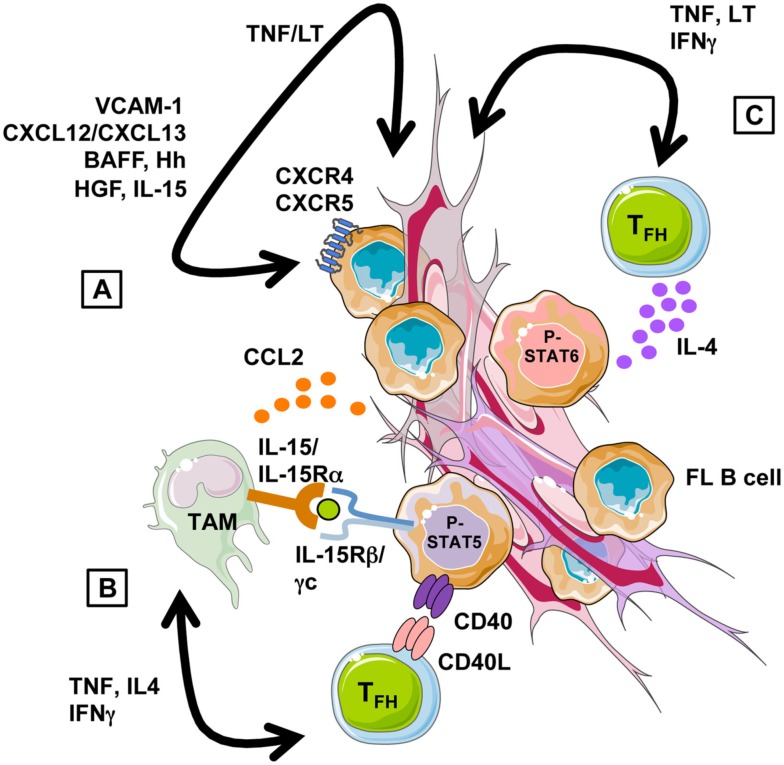
**Follicular lymphoma cell niche**. **(A)** Stromal cells recruit and support directly the growth of FL B cells through a combination of adhesion molecules, chemokines, and cytokines. Their supportive properties are strongly influenced by the local cytokine context, in particular by TNF-α and LT-α1β2 that are overexpressed by malignant B cells and promote lymphoid stromal cell differentiation. **(B)** FL-MSC overexpress the chemokine CCL2 that promotes monocyte recruitment and polarization into TAM-like cells. FL-TAM overexpress IL-15 that triggers, in cooperation with TFH-derived CD40L, STAT5-dependent FL B cell activation. **(C)** FL-TFH overexpress TNF-α and LT-α1β2, which favor stromal cell engagement into FRC differentiation, but also IL-4 that drive STAT6-dependent B-cell activation and contribute to the induction of TAM phenotype.

Interplay between lymphoma cells and their microenvironment provides pivotal signals for malignant cell recruitment and growth (Figure 2). First, stromal cells have been involved in the homing of FL B cells that display a CXCR4^hi^CXCR5^hi^CCR7^lo^ phenotype, resembling to normal GC B cells (Lopez-Giral et al., 2004). We have in particular identified the CXCR4-ligand CXCL12 as the central chemokine in the recruitment of FL cells by both BM and LN-derived stromal cells (Amé-Thomas et al., 2007). Interestingly, CXCL13 has an additive effect with CXCL12 for the migration of FL cells *in vitro* (Husson et al., 2002) and these two chemokines, both produced by FDC *in vivo*, could contribute to the follicular localization of malignant cells. In the Eμ-Myc transgenic mouse model, CCR7 was elegantly reported to drive the specific homing of malignant B cells to the T cell zone where their promote the formation of a lymphoma-supportive stromal cell niche (Rehm et al., 2011). The potential role of CCR7 in human FL is less clear, given its low expression on malignant clones. However, we recently underlined using Affymetrix microarrays that *CCR7* expression is low but upregulated in FL cells compared to normal centroblasts and centrocytes (our unpublished data). The cues that guide normal GC B cell migration have been recently described in mice and involve fine coregulation of CCR7, sphingosine 1-phosphate (S1P) receptor type 2 (SIPR2, also called S1P2), and Epstein-Barr virus-induced gene 2 (EBI2, also known as GPR183) whose ligands are produced by various lymphoid stromal cell subsets. CCR7 is the receptor for CCL19/CCL21 produced by FRC of the T cell zone. SIPR2 belongs to the family receptors for S1P, a lipid signaling molecule presents in a decaying gradient in the follicle. S1PR2 engagement by S1P inhibits cell migration and is involved in the retention of GC B cells. Finally, the EBI2 ligand 7α,25-dihydroxycholesterol is suggested to be present at high levels in outer follicle and interfollicular regions. Briefly, normal antigen-activated B cells upregulate CCR7 and EBI2 and localize to the T-B border zone before migrating to the outer follicle region. By day 4, the downregulation of CCR7 and EBI2, and the upregulation of S1PR2 direct their movement and their confinement to the follicle center, allowing GC formation (Pereira et al., 2010; Green and Cyster, 2012). Interestingly, *S1PR2* is downregulated in FL whereas *EBI2* is similarly expressed in FL cells and normal GC B cells (our unpublished results). How the specific migratory profile of FL B cells compared to their normal counterpart, i.e., similarly high expression of CXCR4 and CXCR5, similarly low expression of EBI2, higher expression of CCR7, and lower expression of S1PR2, could be involved in their dissemination pattern is a fascinating issue. In addition, even if the mechanisms of S1PR2 lymphoma suppressor function remain to be elucidated, S1PR2-deficient mice develop tumors displaying histologic and molecular features of GC-derived DLBCL and *S1PR2* is aberrantly mutated in 26% of human DLBCL (Cattoretti et al., 2009).

Besides migration, stromal cells provide essential survival factors to FL B cells. Among them, hedgehog (Hh) ligands are secreted by BM and LN stromal cells, including FRC and FDC, and prevent spontaneous normal and malignant GC B cell apoptosis (Sacedon et al., 2005; Dierks et al., 2007). In addition, paracrine Hh signaling induces the upregulation of the drug transporter ATP-binding cassette (ABC) G2 and could be involved in stroma-mediated chemotolerance in indolent lymphomas (Singh et al., 2010). The gain of cell-autonomous activation of Hh pathway in DLBCL compared to FL, likely contributes to stroma-independence in this aggressive disease (Kim et al., 2009). Drug resistance has also been associated to the induction by stromal cells of microRNA-181a in malignant B cells, promoting a downregulation of the proapoptotic protein Bim (Lwin et al., 2010). However, whereas regulation of miRNA expression in lymphoma cells by stromal cells is probably a more common event than previously anticipated (Lin et al., 2011), the mechanisms of this modulation remain unsolved. VLA-4-dependent adhesion to stromal cells also protects FL B cells from apoptosis induced by therapeutic antibodies, in particular the anti-CD20 antibody rituximab (Mraz et al., 2011). Interestingly, the VLA-4-ligand VCAM-1/CD106 is upregulated during lymphoid stroma differentiation with a very strong expression in FDC. BAFF, IL-15, and HGF are also produced by FDC and have been proposed to contribute to the antiapoptotic effect of stromal cells on normal and malignant GC B cell growth (Park et al., 2004; Tjin et al., 2005, 2006; Mueller et al., 2007; Lwin et al., 2009; Epron et al., 2012), whereas the role of FDC-derived Wnt5a and Notch ligands in GC-derived lymphomas has not been explored to date (Yoon et al., 2009; Kim et al., 2012).

Importantly, neoplastic B cell-stroma interaction should really be considered as a bidirectional crosstalk. We have previously demonstrated that human secondary lymphoid organs contain *bona fide* mesenchymal stromal cells (MSC) that could be committed, like BM-MSC, to FRC differentiation in response to a combination of tumor necrosis factor (TNF)-α and lymphotoxin (LT)-α1β2, the two main factors involved in the differentiation and maintenance of secondary lymphoid organs (Amé-Thomas et al., 2007). These FRC-like cells are more powerful than resting MSC to support malignant B cell survival. Moreover, malignant B cells themselves could trigger such FRC engagement, at least in part through the release of high levels of TNF-α and LT-α1β2 (Guilloton et al., 2012). In agreement, MSC obtained from invaded BM of FL patients (FL-MSC) are already committed to a FRC-like differentiation, and support more efficiently the growth of malignant B cells than MSC obtained from BM of healthy donors (HD-MSC). FL-MSC display a specific gene expression profile (Guilloton et al., 2012) and it would be of major importance to identify the mechanisms of their increased direct tumor-promoting capacity.

Finally, the activity of stromal cells on malignant B cells could be modulated depending on the cytokine context. We have demonstrated that human MSC, in particular after commitment to a FRC-like phenotype, could express indoleamine-2,3 dioxygenase in response to interferon (IFN)-γ signaling, leading to an inhibition of normal and malignant B cell proliferation but not survival (Maby-El Hajjami et al., 2009). Of note, *IFNG* is upregulated within FL microenvironment, compared to reactive non-malignant LN. Similarly, mice FRC could express nitric oxide synthase 2 after priming by IFN-γ and could thereafter inhibit T cell proliferation to control T cell expansion (Lukacs-Kornek et al., 2011; Siegert et al., 2011). In addition, it has been suggested that adhesion of lymphoma B cells to BM stroma could induce a p27-dependent reversible cell cycle arrest, promoting cell survival, and drug resistance (Lwin et al., 2007). Finally, stromal cells are well known to secrete tumor-transforming growth factor (TGF)-β that inhibits the proliferation of GC-derived B cell lines in a Smad1-dependent manner (Munoz et al., 2004). Altogether these data demonstrate that stromal cells finely regulate the behavior of lymphoma B cells that, in turn, affect their differentiation and activation.

## Indirect Protumoral Activities of Stromal Cells

Besides this direct lymphoma-supportive activity, stromal cells could also interact with non-malignant tumor-infiltrating cells and orchestrate FL cell niches. We have in particular demonstrated that FL-MSC overexpress CCL2 at both RNA and protein levels compared to HD-MSC (Guilloton et al., 2012). Interestingly CCL2 is detectable at higher levels within FL-invaded BM compared to normal BM. Moreover, CCL2 is upregulated in HD-MSC by coculture with malignant B cells in a TNF-α-dependent manner. Tumor-derived CCL2 recruits monocytes in several malignant models (Roca et al., 2009; Qian et al., 2011). We have demonstrated that CCL2 specifically contributes to monocyte recruitment by FL-MSC that in turn trigger their differentiation toward a proangiogenic and anti-inflammatory TAM-like phenotype (Guilloton et al., 2012). Furthermore, stromal cells and macrophages cooperate to sustain malignant B cell survival and proliferation *in vitro*. The reverse activity of macrophages on MSC remains to be evaluated in this context. The poor prognostic value of a high TAM content in FL patients treated with conventional chemotherapy has been reproducibly documented (Relander et al., 2010). However the precise mechanism of the supportive activity of macrophages toward neoplastic B cells remains unknown. Among the candidates, we recently demonstrated that purified FL-TAM overexpress IL-15 that cooperates with T cell-derived CD40L signal to sustain FL cell growth (Epron et al., 2012). In DLBCL, BAFF was also proposed as a monocyte-derived survival factor (Mueller et al., 2007). Another highly interesting signaling pathway is the expression of C-type lectins by myeloid cells that could crosslink the mannosylated FL BCR independently of antigen specificity (Stevenson and Stevenson, 2012).

Another crucial FL-supportive cell subset is the CD4^pos^ CXCR5^hi^ICOS^hi^PD-1^hi^ T_FH_ compartment. T_FH_ provide survival signals to antigen-selected normal GC B cells and help them to achieve class-switch recombination and terminal differentiation into antibody-secreting plasma cells (Crotty, 2011). In human, T_FH_ are also a major source of CXCL13 within follicles. We have first demonstrated that T_FH_ could be found in very high numbers within the FL cell niche and efficiently support the survival of FL cells *in vitro*, unlike non-TFH CD4^pos^ T cells (Amé-Thomas et al., 2012). In agreement, FL-T_FH_ overexpress several genes directly involved in B cell activation, in particular *CD40LG* and *IL4*, and we confirmed the importance of the T_FH_-dependent IL4 centered pathway (Pangault et al., 2010). Interestingly, compared to tonsil-T_FH_, FL-T_FH_ also overexpress some genes involved in the crosstalk with stromal cells, in particular *TNF* and *LTA*, that could favor the differentiation and maintenance of lymphoid stromal cells and the production of CCL2. Lymphoid stromal cells favor T_FH_ survival *in vitro* (our unpublished results) suggesting another positive loop that sustains lymphomagenesis.

Follicular lymphoma cell niche should be thus considered as a highly intricate network of heterogeneous cell subsets where stromal cells directly promote tumor growth but play also a central role as global organizers of the malignant microenvironment through the regulation of non-B cell recruitment, survival, and polarization.

## Stromal Cells as Therapeutic Targets in Lymphoma

Given the preeminent role of stromal cells within FL microenvironment, they progressively emerged as promising therapeutic targets in this essentially incurable disease. A first approach is to transiently mobilize malignant B cells outside their protective niches to render them more sensitive to chemo- and immunotherapy. CXCR4 antagonists, such as plerixafor (AMD3100), could be combined with drugs targeting the malignant cells, such as anti-CD20 antibodies, to increase their activity (Hu et al., 2012). Cell-penetrating lipidated peptides targeting CXCR4 intracellular domains also significantly increase the antitumoral activity of rituximab *in vitro* and *in vivo* (O’Callaghan et al., 2012). Anti-VLA-4 antibody natalizumab has been evaluated as an alternative stromal adhesion-disruptive drug (Mraz et al., 2011). Finally lenalidomide, an immunomodulatory drug clinically active in several mature B cell malignancies and under evaluation in FL, could disrupt B cell-stroma interaction through a decrease in both CXCL12 production by stromal cells (Wobus et al., 2012) and RhoH expression in malignant B cells (Troeger et al., 2012). Interestingly, the Btk inhibitor PCI-32765 has been initially developed to target BCR signaling but it also impairs the chemokine-induced adhesion and migration of primary chronic lymphocytic leukemia B cells (de Rooij et al., 2012). Similar results have been obtained with Syk and PI3K inhibitors and a recent study suggests that the Syk-mTOR pathway is involved in FL cell invasion, through the regulation of metalloproteinase-9 (MMP-9) and angiogenesis, as an upstream regulator of vascular endothelial growth factor (VEGF; Fruchon et al., 2012). Collectively, these results demonstrate that these antagonists of BCR-related kinases could also affect lymphoma cell homing and retention within specific niches. Surface molecules, growth factors, or signaling pathways involved in the bidirectional crosstalk between malignant B cells and stromal cells also represent individual attractive therapeutic targets even if the causative lymphoma-driven signals remain to be precisely defined.

## Concluding Remarks

*In vitro* and *in vivo* studies corroborate the hypothesis that lymphoid BM and LN stromal cells play a central role in the development, growth, progression, and drug resistance in FL. The mechanisms of the crosstalk between malignant B cells and stromal cells are currently the subject of intensive research and several major questions remain unsolved. Whether or not the concept of cancer stem cells applies to this disease and, if true, what are their specific cell niches remains to be explored and could have major therapeutic consequences. Another important issue is the specificity of FL cell niches. Are they shared with premalignant follicular-lymphoma like cells or are they progressively induced by the contact with malignant cells? A detailed comparison of stromal cell niches in BM versus LN and their longitudinal study at diagnosis in the context of minimal residual disease and in relapse would be helpful to define the minimal but essential cell components that support FL cells and rescue them from drug cytotoxicity. It has also to be understood how such a supportive stromal microenvironment is assembled, in particular outside LN. The exact origin of ectopic FRC and FDC, and their relationships with local MSC should be further analyzed. Finally, developing new tools to better understand the functional heterogeneity of FL stroma may provide innovative strategies to disarm the vicious circle where stromal cells support malignant B cells that in turn convert their niche into a fully supportive microenvironment.

## Conflict of Interest Statement

The authors declare that the research was conducted in the absence of any commercial or financial relationships that could be construed as a potential conflict of interest.
